# Patients Admitted for Variant Alpha COVID-19 Have Poorer Outcomes than Those Infected with the Old Strain

**DOI:** 10.3390/jcm10163550

**Published:** 2021-08-12

**Authors:** Matteo Vassallo, Sabrina Manni, Camille Klotz, Roxane Fabre, Paola Pini, Elea Blanchouin, Audrey Sindt, Laurene Lotte, Jean Marc Dubertrand, Stephane Liguori, Nathalie Berkane, Yannick Duval, Fabien Rolland, Christian Pradier

**Affiliations:** 1 Department of Internal Medicine/Infectious Diseases, Cannes General Hospital, 06400 Cannes, France; s.manni@ch-cannes.fr (S.M.); klotz.c@ch-cannes.fr (C.K.); paolapini@yahoo.it (P.P.); e.blanchouin@ch-cannes.fr (E.B.); 2 Department of Public Health, Nice University Hospital, University Côte d’Azur, 06202 Nice, France; fabre.r@chu-nice.fr (R.F.); pradier.c@chu-nice.fr (C.P.); 3 Cognition Behaviour Technology Research Unit, Memory Center, Université Côte d’Azur, 06108 Nice, France; 4 Multipurpose Laboratory, Bacteriology and Virology Unit, Cannes General Hospital, 06400 Cannes, France; a.sindt@ch-cannes.fr (A.S.); l.lotte@ch-cannes.fr (L.L.); 5 Bioesterel Biogroup, 06400 Cannes, France; jeanmarc.dubertrand@biogroup.fr; 6 Multipurpose Laboratory, Bacteriology and Virology Unit, Antibes General Hospital, 06100 Antibes, France; stephane.liguori@ch-antibes.fr; 7 Department of Cardiology, Cannes General Hospital, 06400 Cannes, France; n.berkane@ch-cannes.fr; 8 Department of Pneumology, Cannes General Hospital, 06400 Cannes, France; y.duval@ch-cannes.fr (Y.D.); f.rolland@ch-cannes.fr (F.R.)

**Keywords:** COVID-19, pneumonia, variant alpha, clinical outcome, comorbid conditions

## Abstract

Objectives: The variant alpha COVID-19 rapidly spread across Europe in early 2021. While this variant’s increased infectivity has been proven, little is known of its clinical presentation and outcomes compared to the old strain. Methods: We identified patients admitted to the Cannes General Hospital for variant alpha-related COVID-19 infection from January to April 2021. Their main demographic parameters, inflammatory markers and clinical characteristics were recorded. Patients admitted from October to December 2020 for 20E (EU1) COVID-19 were selected as controls. Differences between groups were analyzed. Results: We included 157 patients (mean age 73 years; 58% men; mean delay of symptoms 6.9 days). Comorbidities were present in 92% (mainly hypertension, diabetes and obesity or overweight). The prevalence of comorbidities did not differ between groups. In 28% of cases, patients either died or required transfer to the Intensive Care Unit (ICU). The cause of death or of transfer to the ICU was presumably associated with severe pneumonia. Variant alpha COVID-19 had 3.8-fold higher risk of death or transfer to the ICU compared to the old strain. Discussion: Patients infected with variant alpha COVID-19, despite similar background characteristics, had a higher risk of unfavorable outcomes than those infected with the old strain, suggesting increased virulence related to this variant.

## 1. Introduction

The current SARS-Cov-2 (Severe Acute Respiratory Syndrome Coronavirus 2) pandemic (COVID-19) has continued to spread worldwide [1,2,3], acquiring mutations in the process, and new SARS-Cov-2 variants were identified in December 2020 with potentially higher infectivity, thus raising concerns regarding immune escape and vaccine efficacy [4].

Among these, the variant alpha (20I/501Y.V1) became rapidly prevalent across Europe in early 2021 [5,6,7] and is now the dominant strain in France, accounting for over 90% of current infections in the Southeastern region of the country [8]. While the clinical presentation of the old strain has been widely described [1,2,3,9], little is known of the clinical characteristics and outcomes of patients infected by the variant alpha. While Challen et al. found higher risks of mortality, Frampton et al. confirmed the increased transmissibility but not the severity [10,11].

The aim of this study was to collect the clinical and demographic characteristics of patients admitted to a medical department for COVID-19 due to the variant alpha and to compare these data with those of patients admitted for COVID-19 due to the old strain (20E (EU1)) during the previous wave which swept through our region in the last quarter of 2020.

## 2. Methods

### 2.1. Study Design and Participants

We conducted an observational and retrospective cohort study of patients admitted to a medical department in Cannes General Hospital from January to April 2021 with confirmed variant alpha COVID-19 infection. These were compared to patients admitted to the same department for COVID-19 due to the 20E (EU1) strain between October and December 2020. Data from each patient group were extracted from the hospital electronic database. During this period, there was a major second outbreak of COVID-19 in our region, while the first wave had lasted from March to May 2020 [12].

Patients with positive SARS-CoV-2 RT-PCR detected in nasal swabs and screening samples for either 20I/501Y.V1 or the 20E (EU1) strains were included.

The study was submitted to the Health Data Hub (https://www.health-data-hub.fr/depot (accessed on 26 May 2021), and all patients provided written informed consent.

The patient demographics; underlying comorbidities; duration of symptoms; clinical signs prior to admission, upon admission and during hospitalization; laboratory findings during hospital stay; management; and clinical outcome were collected from their medical records. Since excess weight was also a suspected risk factor for severe COVID-19 [13,14,15], it was included among comorbidities by using the WHO definitions for adults, i.e., patients with Body Mass Index (BMI) between 25 and 30, while for values above 30, patients were considered obese [16]. 

In line with the WHO criteria for COVID-19 clinical severity [17], patients were divided into the following groups.

(1) Mild to moderate disease (mild symptoms up to mild pneumonia).

(2) Severe disease (dyspnea, hypoxia or over 50% lung involvement as shown on the CT scan).

Patients with critical illness severity upon arrival and admitted directly to the Intensive care unit (ICU) were not included.

In the case of patient transfer to another clinical department, files were reviewed and physicians were interviewed in order to confirm that no significant changes in clinical presentation had been observed for each patient.

According to clinical outcome, two patient groups were defined: Those who recovered during their hospital stay and those who either died or required transfer to the ICU. The causes of death or transfer to the ICU were also investigated.

### 2.2. Laboratory Markers

The following measurements were collected and included in statistical analysis: C-reactive protein; white cell; neutrophil; hemoglobin and platelets counts; D-dimer; and IL-6 (electrochemiluminescence immunoassay, Cobas^®^ 6000 Roche, pg/mL). RT-PCR assays were either performed in the multipurpose laboratory of Cannes General Hospital or in the Bioesterel Biogroup laboratory. According to the study period and to the laboratory guidelines, four RT-PCR assays were used: the NeuMoDX^®^ (Qiagen) instrument (targeting N and NSP2 genes), the GeneXpert^®^ (Cepheid, E and N2 genes), the Gene Finder COVID-19 Plus RealAmp kit (GeneFinder, N, E and RdRP genes) or the SARS-Cov-2 ELITe MGB kit (Elitech, ORF8 and RdRP genes). Screening of virus isolate was realized by targeting N501Y, E484K and K417N mutations using the GSD NovaType II SARS CoV-2 and NovaTec Immundiagnostica GmbH assays.

### 2.3. Statistical Analysis

The main demographic, laboratory and clinical characteristics and the clinical outcomes of patients infected with the variant alpha were compared with those of patients infected with the old strain by using χ^2^-tests or Fisher’s exact test and Student′s *t*-tests or the Wilcoxon–Mann–Whitley test. The independent risk factors associated with the variant alpha were identified by logistic regression. 

Moreover, by using univariate and multivariate models, we analyzed also risk factors associated with a poor clinical outcome, defined by either death or transfer in ICU. The variables with a *p*-value ≤ 0.20 in the univariate analysis were initially selected for the multivariate model, and only those with a *p*-value ≤ 0.05 were retained in the final model.

Lastly, Spearman’s rho correlations were calculated for the laboratory markers. We also included in this analysis the neutrophil to lymphocyte and the platelet to lymphocyte ratios, which have been shown to be predictive biomarkers of severity in COVID-19 [18,19]. All analyses were performed by using the R 4.0.5 software.

## 3. Results

### 3.1. Patient Characteristics

From January to April 2021, 65 patients with variant alpha COVID-19 infections were admitted to the medical department in Cannes General Hospital. These were compared to a control group of 93 patients admitted for 20E (EU1) COVID-19 infection between October and December 2020. They all came from the geographic area of Cannes and its neighborhoods. 

The analysis was thus conducted on 158 patients (mean age 73 years; mean delay since symptoms onset: 6.9 days). No patient had been immunized against COVID-19 prior to admission. Ninety-two percent of patients presented with comorbidities, and most of these were hypertensive, diabetic, obese or overweight.

Variant alpha COVID-19 was identified more often among female patients, while the prevalence of comorbid conditions did not differ between groups.

### 3.2. Laboratory Markers

Platelets counts and platelet to lymphocyte ratios were higher in the variant alpha group (Table 1). 

Spearman’s rho showed that inflammatory biomarkers were highly correlated among them. In particular, Il-6 levels were significantly associated with the other markers of inflammation, including CRP (see Supplementary Table S1).

### 3.3. Clinical Outcome

Standard of care for all patients included anticoagulant therapy and steroids were systematically prescribed for those with severe pneumonia, generally consisting of dexamethasone 0.1–0.2 mg/kg/day for 10 days. Only two patients received the anti-IL6 agent Tocilizumab. 

In 44 out of 158 cases (28%), patients either died or were transferred to the ICU. Prevalence of death was slightly higher in the variant alpha group (15.4% vs. 12.9%) and the main difference was represented by the increased number of transfers in the ICU in the variant alpha group (27.7% vs. 8.6%).

The causes of death or transfer were all presumably associated with increased severity of pneumonia. Moreover, among subjects transferred in ICU, four patients in the variant alpha group unfortunately died, and three in the 20E (EU1) group also died. Differences were not statistically significant (data not shown).

Fatal outcomes or transfers to the ICU were significantly more frequent among patients with the variant alpha than among those with the 20E (EU1) strain (Table 1). 

Il-6 and CRP values upon admission, severe pneumonia, the highest oxygen flow value and infection with the variant alpha were associated with either death or transfer to the ICU in univariate analysis (Table 2).

Multivariate analysis showed that patients with the variant alpha COVID-19 had a 3.8-fold higher risk of death or of transfer to the ICU (Table 3). Moreover, the female sex was confirmed to be associated with the variant alpha together with the platelet count, although the OR was 1 (Table 3). Moreover, although the variant alpha preferentially affected women, we found no correlation between sex ratio and poor outcome (Table 4).

The multivariate logistic regression confirmed that the infection with the variant alpha, the severity of hypoxemia and the platelets count were independent risk factors for an unfavorable outcome (Table 4). 

## 4. Discussion

We described the clinical characteristics of patients admitted in a medical department for variant alpha COVID-19 infection. To our knowledge, this is one of the largest clinical cohorts of patients described with this variant, which is at present largely dominant in Europe.

Although the prevalence of comorbidities and age distribution were similar between groups and the geographic area of provenience did not change, the risk of death or of transfer to the ICU was almost four-fold higher for patients with variant alpha COVID-19 compared to patients admitted for COVID-19 due to the old strain during the previous outbreak.

The reasons for such a poorer outcome are still unclear. Challen et al. suggested that either a more advanced stage of disease or comorbid conditions could be responsible for such outcomes [10]. However, as the main demographic parameters, the duration of symptoms and the inflammatory markers did not differ between groups; we suggest that the variant alpha strain could be more virulent than the old strain. Indeed, the emergence of variants with mutations in the S glycoprotein, especially in the receptor binding domain, could not only be associated with enhanced infectivity but also with higher affinity for the Angiotensin-converting enzyme 2 (ACE-2), with a potential for immune escape [4]. Further studies should clarify whether such mutations could confer a higher risk for severe disease. 

Moreover, Thorne et al. recently showed that the variant alpha may increase in vivo replication and duration of the infection by upregulating the expression of key innate immune antagonists [20]. Moreover, although different techniques for RT-PCR were used for our patients, we found that viral loads, measured according to the RT-PCR cycle threshold values in nasal swabs, were higher in the variant alpha group (data not shown). These results are in line with those reported by Davies et al. [21], Challen et al. [10] and Patone et al. [22] but differed from Frampton et al. who, despite observing higher viral loads, found no association with disease severity [11]. Moreover, in line with the results from Patone et al. the worse outcome in the variant alpha group cannot be explained by time-dependent factors as the duration of symptoms were similar and the availability of ICU bed did not significantly change between groups.

Comorbidities were highly prevalent, with many hypertensive patients in both groups. SARS-Cov2 impairs the renin-angiotensin-aldosterone system by binding with ACE-2. The degradation of the enzyme causes increased levels of angiotensin-2, with potential damage to the lungs and also to other tissues due to its pro-inflammatory properties [23]. In hypertensive patients whose angiotensin-2 levels are already increased, such an inflammatory response could be heightened, thus explaining the frequency of this comorbidity. Indeed, in the present study, hypertensive patients had higher levels of IL-6 (data not shown). Obesity and overweightness were also highly prevalent. The reasons for such increased risks could be linked to the expression of ACE-2 receptors in adipose tissue, as suggested by Favre et al. [24]. Heightened Angiotensin-2 production could thus contribute to macrophage activation and polarization resulting in a pro-inflammatory phenotype, potentially triggering the so-called “cytokine storm” observed in severe forms of the disease [25]. In line with the high prevalence of obesity or overweightness in our cohort, France has extended its vaccine guidelines to include young individuals with these conditions among the priority categories for vaccination [26]. Finally, diabetes has also been associated with higher risks of severe COVID-19, probably as a consequence of an imbalance between the lymphocyte T helper 1 and T helper 2 cytokine ratio together with peripheral lymphocyte CD8+ T cell and NK cell dysregulation [27]. 

Although this is a retrospective study including a limited number of patients, files were carefully reviewed, and the authors of this report were also the clinicians who took care of these patients. Unfortunately, complete data describing the prevalence of variant alpha strains also harboring the E484K or the E484Q spike mutations were available only since the end of January, i.e., approximately one month after the variant alpha spread in France. These mutations have shown to promote escape from neutralizing antibodies in vitro, and their clinical presentation characteristics are lacking [28]. However, their prevalence in France is still very low (1.2%) [28], and they have never been found in the few patients of our cohort who benefited from such screening.

## 5. Conclusions

We showed that patients with variant alpha COVID-19 had less favourable outcomes than those with the previous strain, suggesting its higher virulence and warranting further prospective studies.

## Figures and Tables

**Table 1:** Comparison between patients with variant alpha and wild-type COVID-19.

	Variant Alpha	20E (EU1) Strain	*p*-Value
	N (%) or Mean (SD)	N (%) or Mean (SD)	
Number of patients	65	93	
Demographic and background parameters			
Male gender	31 (48%)	60 (65%)	0.035
Age (years)	73.2 (14.9)	72.9 (16.3)	0.888
Age >65 years	49 (75%)	65 (70%)	0.449
Duration of symptoms (days)	6.8 (4.0)	7.0 (4.3)	0.838
Diabetes	15 (23%)	20 (22%)	0.815
Hypertension	33 (51%)	48 (52%)	0.917
Obesity	11 (17%)	15 (16%)	0.918
Overweight	15 (23%)	24 (26%)	0.666
No comorbid conditions	6 (9%)	8 (9%)	0.908
Laboratory markers			
Interleukin-6 at admission * (pg/mL)	92.4 (104.3)	79.5 (86.6)	0.604
C-reactive protein at admission (mg/L)	88.2 (83.6)	102.8 (94.0)	0.345
Neutrophil/Lymphocytes ratio	7.8 (7.2)	7.7 (6.2)	0.922
Platelet/lymphocyte ratio	0.31 (0.24)	0.24 (0.15)	0.007
Platelet count (giga/L)	234.7 (90.5)	207.3 (80.5)	0.039
D-Dimer ** (µg/mL)	1.72 (3.0)	1.75 (3.2)	0.500
Clinical characteristics and outcome			
Severe pneumonia	52 (80%)	63 (69%)	0.088
PaO2 on room air (mmHg) ***	63.3 (16.9)	70.0 (23.9)	0.150
PaO2 on O2 therapy (mmHg) ****	76.8 [47.2]	68.4 (22.1)	0.396
Worst PaO2/FiO2-mean (SD) ****	169.0 [130.6]	157.9 (85.8)	0.710
Death or transfer to the ICU	26 (40%)	18 (19%)	0.004

* *n* = 85; ** *n* = 80; *** *n* = 84; **** *n* = 55; ICU = Intensive Care Unit.

**Table 2:** Factors associated with the clinical outcome.

	Neither Death nor ICU Transfert-*n* = 114		Either Death or ICU Transfert-*n* = 44		
	Mean	(SD)	Mean	(SD)	*p*-Value *
Age	72.60	(16.27)	74.09	(14.41)	0.790
Days of symptoms (*n* = 157)	7.06	(4.26)	6.63	(3.93)	0.572
Il-6 (pg/mL) (*n* = 85)	66.69	(72.70)	118.87	(118.85)	0.020
C-reactive protein (mg/L)	88.22	(88.78)	119.02	(90.02)	0.017
Hemoglobine (g/dL)	13.24	(1.57)	12.75	(1.92)	0.098
Lymphocytes (giga/L) (*n* = 156)	1046.81	(536.88)	885.81	(392.90)	0.060
Platelets (giga/L)	226.27	(87.38)	198.59	(78.04)	0.096
D-Dimers (µg/mL ) (*n* = 80)	1.62	(2.70)	2.00	(3.88)	0.284
N/L ratio (*n* = 156)	7.39	(7.75)	7.42	(4.83)	0.121
P/L ratio (*n* = 156)	0.27	(0.22)	0.26	(0.14)	0.369
	*n*	(%)	*n*	(%)	*p*-value **
Viral strain					0.004
20E (EU1)	75	(65.8)	18	(40.9)	
Variant alpha	39	(34.2)	26	(59.1)	
Sex					0.189
Female	52	(45.6)	15	(34.1)	
Male	62	(54.4)	29	(65.9)	
Hypertension					0.386
No	58	(50.9)	19	(43.2)	
Yes	56	(49.1)	25	(56.8)	
Diabetes					0.750
No	88	(77.2)	35	(79.5)	
Yes	26	(22.8)	9	(20.5)	
Age > 65 years					0.198
No	35	(30.7)	9	(20.5)	
Yes	79	(69.3)	35	(79.5)	
Max oxygen flow					<0.001
<3 L/min	104	(91.2)	14	(31.8)	
>3 L/min	10	(8.8)	30	(68.2)	
Either overweight or obesity					0.431
No	70	(61.4)	24	(54.5)	
Yes	44	(38.6)	20	(45.5)	
Clinical Severity					<0.001
Mild to moderate	42	(36.8)	1	(2.3)	
Severe	72	(63.2)	43	(97.7)	

N/L: neutrophil to lymphocyte; P/L: Platelet to lymphocyte. *: Student T test or Wilcoxon Mann Whitney test. **: Chi square test or Fisher’s exact test.

**Table 3:** Risk factors associated with the variant alpha.

	Risk Factors Associated with the Variant Alpha		
	AdjOR	(95% CI)	*p*-Value
Platelet count	1.00	(1.00; 1.01)	0.023
Death or transfer in ICU			
No	1		
Yes	3.77	(1.77; 8.36)	<0.001
Sex			
Female	2.25	(1.13; 4.55)	0.022
Male	1		

**Table 4:** Risk factors for either death or transfer in the ICU.

	Risk Factors for either Death or Transfer in ICU		
	AdjOR	(95% CI)	*p*-Value
Platelet count	0.99	(0.98; 0.99)	0.004
Max Oygen flow (l/min)	1.68	(1.39; 2.14)	<0.001
Viral strain			
20E (EU1)	1		
Variant alpha	3.18	(1.30; 8.10)	<0.001

ICU: Intensive Care Unit.

## Data Availability

The original contributions presented in the study are included in the manuscript; further inquiries can be directed to the corresponding authors.

## Supplementary Materials

The following are available online at https://www.mdpi.com/article/10.3390/jcm10163550/s1. Table S1: Spearman’s rho correlations for laboratory markers.
