# Supplementary material for: Patients Admitted for Variant Alpha COVID-19 Have Poorer Outcomes than Those Infected with the Old Strain
Source: J Clin Med. 2021 Aug 12;10(16):3550. doi: 10.3390/jcm10163550 (PMC8396910; doi:10.3390/jcm10163550)
Supplement: Supplementary file 1 [file jcm-10-03550-s001.zip › jcm-1292372-supplementary.pdf]

Table S1: Spearman's rho correlations for laboratory markers.

		Il-6	CRP	Leucocytes	Neutrophiles	Hemoglobine	Lymphocytes	Platelets	D-dimers	N/L ratio
CRP	Spearman's Rho	**0.40**	-	-	-	-	-	-	-	-
	P-value	**<0.001**	-	-	-	-	-	-	-	-
Leucocytes	Spearman's Rho	**0.26**	**0.47**	-	-	-	-	-	-	-
	P-value	**0.015**	**<0.001**	-	-	-	-	-	-	-
Neutrophiles	Spearman's Rho	**0.34**	**0.54**	**0.97**	-	-	-	-	-	-
	P-value	**0.002**	**<0.001**	**<0.001**	-	-	-	-	-	-
Hemoglobine	Spearman's Rho	−0.16	0.04	0.01	0.01	-	-	-	-	-
	P-value	0.150	0.589	0.911	0.911	-	-	-	-	-
Lymphocytes	Spearman's Rho	−0.01	−0.13	**0.20**	0.01	0.01	-	-	-	-
	P-value	0.920	0.113	**0.014**	0.855	0.895	-	-	-	-
Platelets	Spearman's Rho	0.03	0.13	**0.47**	**0.44**	**−0.23**	0.17	-	-	-
	P-value	0.783	0.094	**<0.001**	**<0.001**	**0.004**	0.031	-	-	-
D-dimers	Spearman's Rho	**0,29**	**0.29**	**0.22**	**0.28**	−0.16	−0.04	0.18	-	-
	P-value	**0.040**	**0.009**	**0.052**	**0.012**	0.152	0.711	0.119	-	-
N/L ratio	Spearman's Rho	**0.29**	**0.49**	**0.63**	**0.77**	0.00	**-0.57**	**0.23**	**0.25**	-
	P-value	**0.009**	**<0.001**	**<0.001**	**<0.001**	0.968	**<0.001**	**0.004**	**0.027**	-
P/L ratio	Spearman's Rho	0.03	**0.19**	**0.17**	**0.29**	**−0.19**	**−0.67**	**0.56**	0.19	**0.62**
	P-value	0.785	**0.017**	**0.039**	**<0.001**	**0.017**	**<0.001**	**<0.001**	0.085	**<0.001**

N/L = Neutrophil to Lymphocyte; P/L = Platelet to Lymphocyte
